# Application of Machine Learning Techniques to Help in the Feature Selection Related to Hospital Readmissions of Suicidal Behavior

**DOI:** 10.1007/s11469-022-00868-0

**Published:** 2022-07-18

**Authors:** Gema Castillo-Sánchez, Mario Jojoa Acosta, Begonya Garcia-Zapirain, Isabel De la Torre, Manuel Franco-Martín

**Affiliations:** 1 Department of Signal Theory and Communications, and Telematics Engineering, Universidad de Valladolid, Paseo de Belén 15, 47011 Valladolid, Spain; 2 eVida Research Laboratory, The University of Deusto, Bilbao, Spain; 3 Psychiatry Service, Healthcare Complex, Zamora, Spain

**Keywords:** Machine learning, Readmissions, Mental disorder, Suicide prevention, Hospital

## Abstract

Suicide was the main source of death from external causes in Spain in 2020, with 3,941 cases. The importance of identifying those mental disorders that influenced hospital readmissions will allow us to manage the health care of suicidal behavior. The feature selection of each hospital in this region was carried out by applying Machine learning (ML) and traditional statistical methods. The results of the characteristics that best explain the readmissions of each hospital after assessment by the psychiatry specialist are presented. Adjustment disorder, alcohol abuse, depressive syndrome, personality disorder, and dysthymic disorder were selected for this region. The most influential methods or characteristics associated with suicide were benzodiazepine poisoning, suicidal ideation, medication poisoning, antipsychotic poisoning, and suicide and/or self-harm by jumping. Suicidal behavior is a concern in our society, so the results are relevant for hospital management and decision-making for its prevention.

## Introduction

Suicide was the primary source of death by external causes in Spain in 2020, with 3,941 cases – 7.4% higher than in 2019 (INE, 2021). 4.4% of deaths in Spain corresponded to mental or behavioural disorders (INE, 2021), while in Castilla y León (CYL), there were 228 deaths by suicide in 2020 (INE, 2021).

In CYL, the general trend in psychiatric hospitalizations was an annual statistically significant increase of 2% over 11 years (2005–2015) (Llanes-Álvarez et al., 2021). However, hospitalization in CYL tended to be lower in cases where the main diagnosis was alcohol or drug abuse/dependence (Llanes-Álvarez et al., 2020).

Additional efforts to prevent suicide may better focus on reducing the risk of suicide immediately following discharge (Williams et al., 2018). Mental disorders in themselves do not explain suicide, although it is a fact that there is an underlying mental disorder in most cases and this vulnerability interacts with many psychological and social factors that lead some individuals to either end or try to end their own lives (Haw & Hawton, 2015). For their part, mental disorders are considered major risk factors in suicide (Moitra et al., 2021), with one study suggesting that ongoing efforts are required to improve access to and quality of mental health care, to prevent individuals with mental disorders from committing suicide (Too et al., 2019).

That is why there was a need to gather official information based on the minimum basic dataset (CMBD) (Melendez frigola et al., 2016) regarding admissions or acute patients associated with suicide in CYL, which entails the search for the key factors that have the most bearing on hospital readmissions of such patients with mental health problems by way of the objective to be pursued in this research. Thus, the application of techniques such as CHAID (Jojoa et al., 2021), random forest (Wang et al., 2021), logistic regression (Qasim & Algamal, 2018), and support vector machine (Jojoa-Acosta et al., 2021) proved necessary, together with a set based on common outputs for each algorithm to obtain a general overview of how the resulting system functions. Subsequently, and for the specialist in psychiatry to conduct the assessment, a table was put together indicating which variables best explained the real situation facing each hospital subject to study. Lastly, the results obtained were based on the application of machine learning, conventional statistical methods, and expert assessment provided to suicide prevention strategies, considering the features of each region.

This research presents the materials used in the following section, describing the database used (context-hospital time behavior) and the methods with their basic theoretical principles supporting the selection of machine learning techniques used. The results obtained from the analysis and corresponding comparisons are then provided. Lastly, a discussion of results in considered together with the limitations and conclusions deriving from this research.

## Materials and Methods

### Materials

#### State-of-the-Art Review (Context-Hospital Time Behavior)

CYL (an autonomous region comprising nine provinces (Avila, Burgos, Leon, Palencia, Salamanca, Segovia, Soria, Valladolid and Zamora). It covers an area of 94,226 km2 with 2,409,165 inhabitants as of 2020 (INE, 2021).

**Fig. 1:**
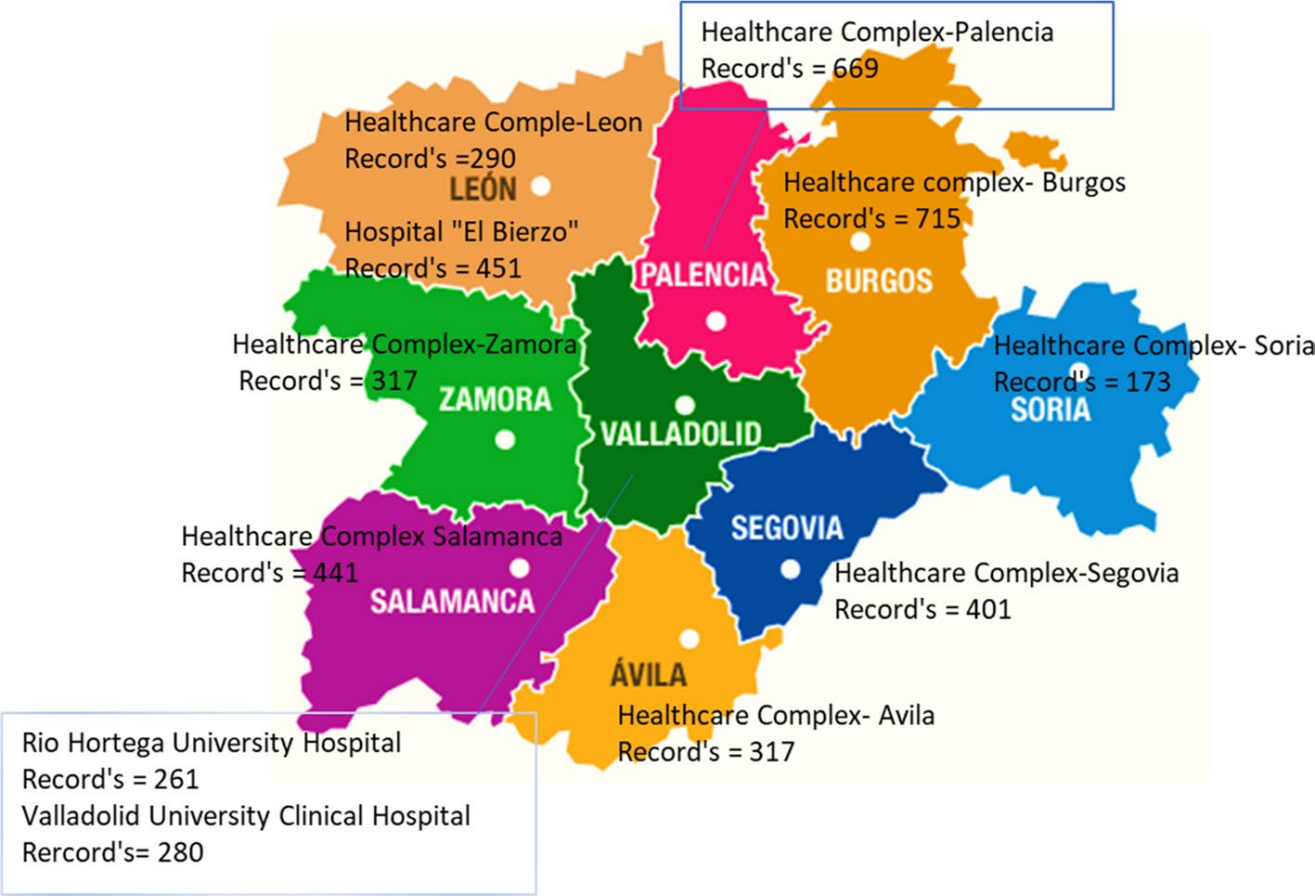
Map of CYL with total numbers of records with suicide-related diagnoses between 2005 and 2015

The distribution of hospitals and their corresponding hospital records of acute patients with suicide-related mental disorders according to the province are shown in Fig. 1.

CYL health organization is based on territorial demarcations (Fig. 1). Figure 1 shows the total number of records of patients with mental disorders who were hospitalized between 2005 and 2015 in CYL.

In Table 1, we can observe the average population corresponding to each hospital region that recorded acute patients with mental health issues.

**Table 1:** Population average over the 11 years (2005–2015): variance, according to region and hospital in CYL

Region	Population average	Standard deviation	Variance
Avila	169,419.4	2576.5	6,638,642.4
Burgos	369,791.4	5526.8	30,546,316.6
El Bierzo Hospital	247,148.3	3424.1	11,724,220.2
Leon	247,148.3	3424.1	11,724,220.2
Palencia	171,286.8	2636.6	6,951,987.5
Salamanca	349,948.5	5114.0	26,153,388.8
Segovia	160,991.2	3444.8	11,866,853.7
Soria	93,739.73	1370.6	1,878,487.8
Valladolid Clinic Hospital	263,986.6	3381.6	11,435,294.4
Zamora	192,909.7	5129.1	26,307,633.2

It is important to highlight the fact that the 261 records about the Rio Hortega hospital in Valladolid were not included, because the gathering of information from this the hospital required for the present study first started to be recorded in 2009, in contrast with the other records that date back to 2005.

The remaining hospitals coincide in the data collection period from 2005 to 2015, and so their 4054 records were included in their entirety for this research.

Furthermore, CYL is a large region in Spain and has a food and agriculture sector with a turnover of around 10% of the rest of Spain, which attention should be drawn to its meat, dairy, and animal foodstuff industry (Invest in Spain, n.d.). Twelve percent of total Spanish energy is produced in CYL, which also boosts energy diversification and innovation in terms of renewable energies (Invest in Spain, n.d.).

#### Dataset Description

Patient admission records in (CYL) comprise 4315 records with diagnoses of acute mental disorders in public health hospitals in Castilla y Leon (SACYL) (Sacyl, 2021) between 2005 and 2015. The data is based on the minimum basic dataset (CMBD) (Melendez frigola et al., 2016) and the International Classification of Diseases 10 (Spain, 2021).

**Fig. 2:**
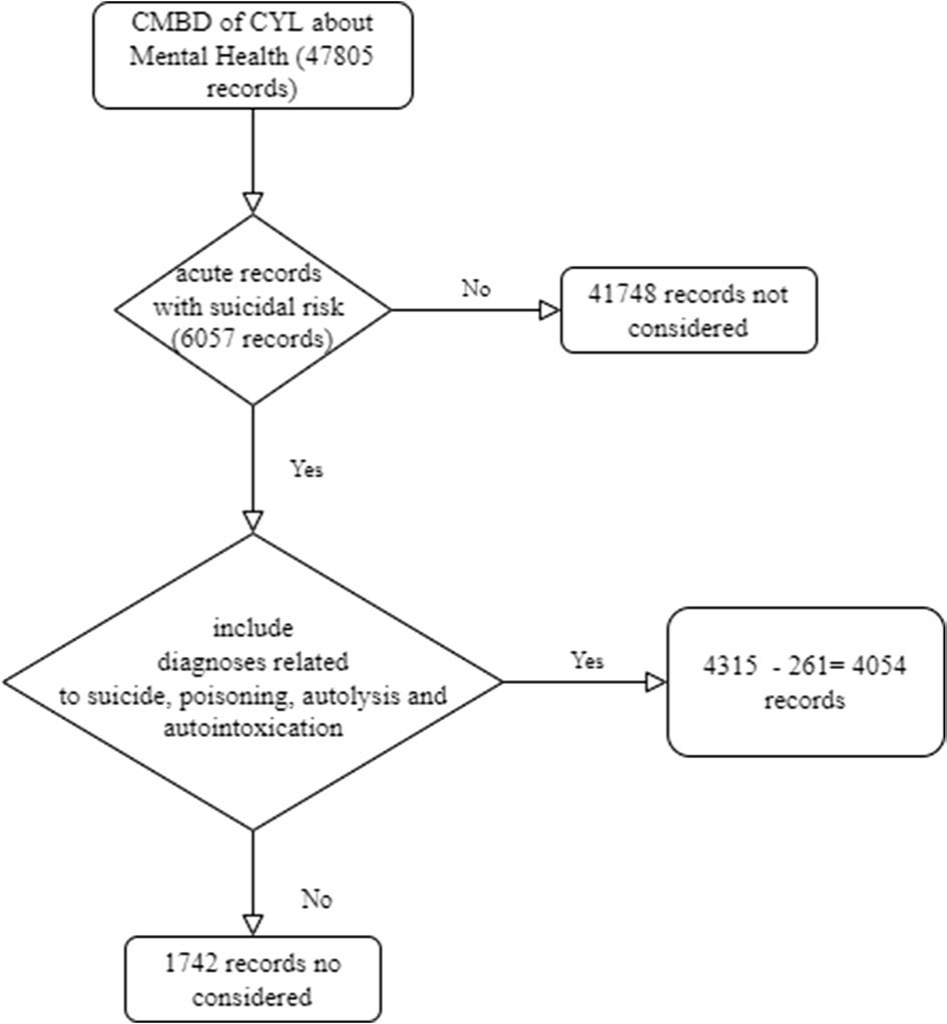
Flow of inclusion and exclusion criteria: patient data associated with suicide-related diagnoses

We applied data cleaning to obtain records of patients with diagnoses associated with suicide, which we show in detail in Fig. 2. The data relating to suicide diagnoses in CYL, which we will refer to as (DBSUICIDECYL), comprises 4315 records of admissions of patients with suicide-related diagnoses. The inclusion criteria for records were acute mental health patients according to ICD-10 coding of the diagnoses selected by the authors as suicide-related disorders shown in Table 2 and Fig. 2. Lastly, *N* = 4054 was used to extract the 261 records from the Rio Ortega University Hospital, according to Fig. 2.

**Table 2:** Variable description of DBSUICIDECYL

*Variable description*		
*Mental disorders*	*Suicidal features*	*Somatic disorders*
**Personality disorder** (includes borderline personality disorder and histrionic personality disorder)	**Antipsychotic poisoning** (includes antipsychotic poisoning)	**Arterial hypertension**
**Bipolar disorder** (includes diagnoses related to manic depressive psychosis)	**Benzodiazepine poisoning**	**Hypercholesterolemia** (includes hyperlipidaemia and lipidaemia)
**Depressive syndrome** (includes depressive disorder)	**Suicide by psychotropics**	**Mellitus diabetes**
**Schizophrenia** (includes schizophreniform, delusional development, and paranoia)	**Drug poisoning** (includes all drug intoxication and suicide by drugs)	**Hypothyroidism**
**Adjustment disorder** (all types of this disorder)	**Suicidal ideas**	
**Alcohol abuse** (includes alcohol dependence and alcohol addiction)	**Suicide and/or self-harm from jumping**	
**Dysthymic disorder**		
**State of anxiety**		

Other variables in the database:

*Years*, a year in which the diagnosis was registered; *Admission month*, patient record admission month; *Hospitals*, Hospital identifier; *Gender*, gender identifier; *Age*, age identifier; *stay days*, number of patient hospital stay days; *Re_entry*, variable assumed value 1 when the patient was readmitted to CYL hospitals during the period from 2005 to 2015

#### Variable Description

Some dichotomous variables are created that allow us to identify the most frequent diagnoses associated with suicide in DBSUICIDECYL, these being organized into three main themes. Other variables such as years are shown numerically, with age and stay days being categorized accordingly together with hospitals. For further details, see Table 2.

Table 3 shows the distribution of mental disorders, suicidal features, and somatic disorders associated with suicide as described by DBSUICIDECYL, according to that shown in Table 2. Distribution according to gender enables us to show the distribution and corresponding percentage in each group of variables included in this study (Table 3).

**Table 3:**
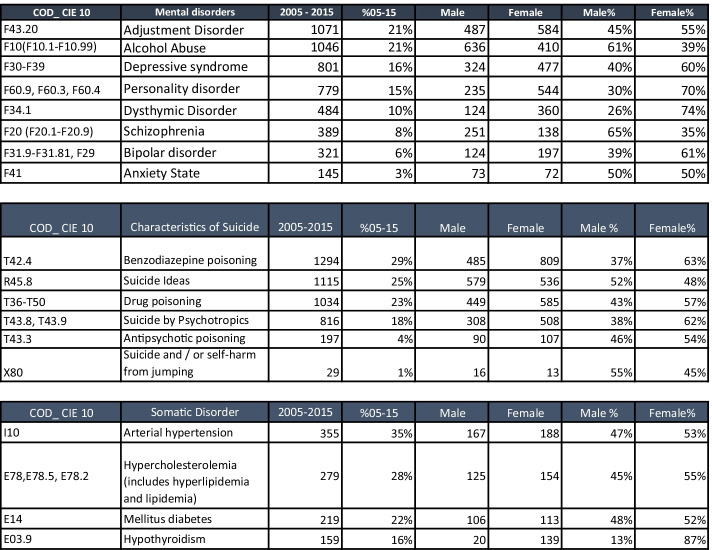
Distribution and percentages of variables according to year and gender, broken down according to mental disorders, suicidal features, and somatic disorders

### Methods

The decision was made in the present research to use two state-of-the-art components in selecting attributes/variables. The first corresponds to a classic statistical technique based on goodness of fit assessed by Chi2 distribution, while the second involves the use of machine learning techniques whose function is based on 3 different approaches: entropy, probability, and the linear ratio of the variable. Based on this, the CHAID algorithms were selected (Jojoa et al., 2021) for the first component, random forest (Wang et al., 2021), logistic regression (Qasim & Algamal, 2018), and support vector machine (Jojoa-Acosta et al., 2021) for the second.

**Fig. 3:**
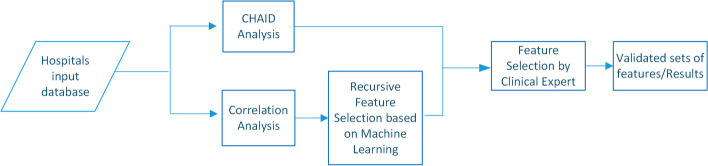
Methods applied

In the end, a study was carried out based on common outputs and assessment by an expert, who finally decided which would have the greatest bearing on the *hospital readmissions* variable from among the resulting set of variables. Details of the methods applied in the present study are provided below in Fig. 3.

#### CHAID Analysis for Feature Selection

The application of different techniques was required for the present study, to compare them and thus obtain target results about those variables that have the greatest bearing on predicting admissions in acute patients with suicide-related mental disorders. To this end, it was important to use a technique based on the statistical study involving the distribution of the data analyzed. Chi-square tests offer the chance to analyze by observing the goodness of fit of one set of data in contrast to the other—in other words, using this method known as chi-square interaction automatic detector (CHAID), it is possible to build a tree that may help to determine how the variables merge to explain the result in the given dependent datum. Nominal, ordinal, and continuous data may be used in the CHAID analysis, in which continuous predictors are divided into categories with approximately the same number of observations. In our case, the response variable evidence dichotomous behavior, enabling the CHAID to detect all the possible cross-tabulations for each categorical predictor until the best result is obtained—exactly where no other division can be made in some branch of the tree. The decision or classification tree starts with the identification of the target variable or dependent variable, which would be considered the root. The CHAID analysis divides the target into two or more categories using statistical algorithms in child nodes. Unlike the regression analysis, the CHAID technique does not require data to be distributed normally.

#### Machine Learning for Feature Selection

We find the use of classification algorithms in many state-of-the-art works which, via information analysis, can identify the most important attributes or variables in a prediction task. That is why we decided to apply different methods based on different linear and-linear metrics and, with the results obtained as a whole, thus determine the importance of attributes when predicting admissions. For this reason, we selected three algorithms with different metrics, as their objectivity was required.

##### Correlation Analysis

In a machine learning analysis, it is desirable for the variables being analyzed not to evidence any correlation with each other, as dimensionality reduction is needed to prevent any phenomena that may affect performance, such as those regarding fit. Therefore, a Pearson correlation coefficient analysis was initially carried out to observe those variables which could be disregarded according to a team of experts.

##### Pearson Correlation Coefficient and Spearman Correlation Coefficient

Two matrixes were created to observe the correlation between input variables: one based on the Pearson correlation coefficient (Wan et al., 2021) and one on the Spearman correlation coefficient (Ghosh et al., 2021).

Initially, we used normalized covariance to thus compare data behavior in the sets being studied, using centred statical moment. The formula corresponding to the Pearson correlation coefficient is shown in Eq. (1)

1 $$\rho_p=\frac{Co(x,y)}{\delta_x\delta_y}\;\mathrm{Pearson}\;\mathrm{correlation}\;\mathrm{coefficient}$$

Seeking a more objective perspective, it was also decided to use the Spearman correlation coefficient [17] in such a way as to ascertain correlation behavior between variables, via the two approaches mentioned:

2 $$\rho_s=1-\frac{6\Sigma\left(x-y\right)^2}{n\left(x^2-1\right)}\;\mathrm{Spearman}\;\mathrm{correlation}\;\mathrm{coefficient}$$

whereas the Pearson correlation coefficient seeks linear correlation between two random variables, the Spearman correlation coefficient targets the monotonous relationship between variables, i.e., they change simultaneously in terms of increase or decrease.

##### Non-correlated Variable Selection

**Fig. 4:**
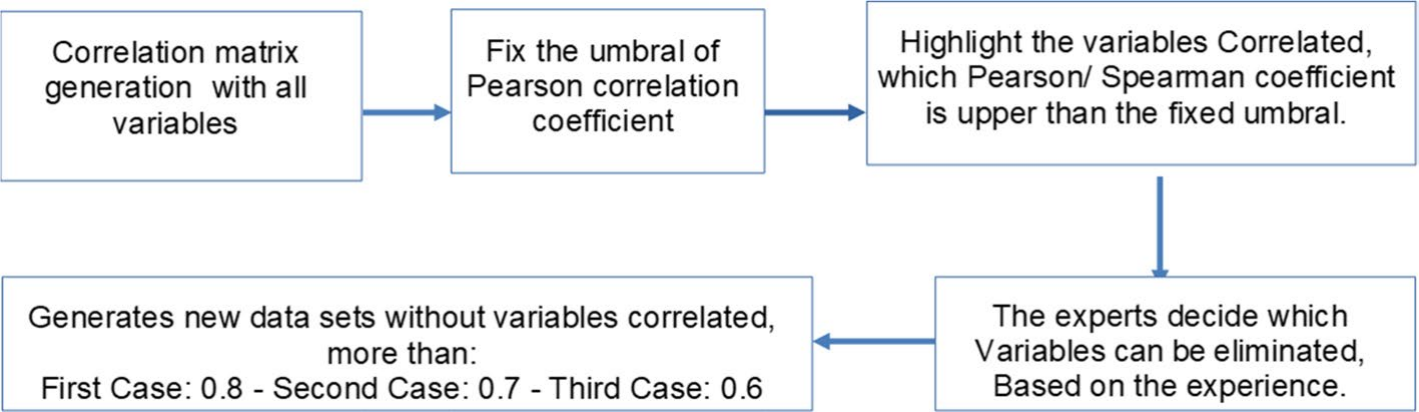
Block diagram showing the creation of subunits based on Pearson and Spearman correlation coefficients

Once the aforementioned procedures have been completed, those variables with a statistically high correlation value are then selected, i.e., those whose correlation coefficients exceed certainly given thresholds. To this end, thresholds of 0.8, 0.7, and 0.6 were established to create non-correlated subunits to select attributes. A block diagram is shown in Fig. 4 with the procedure referred to.

##### Recursive Feature Selection Based on Machine Learning

Currently, machine learning techniques (ML) are being widely used for tasks involving attribute selection (Munasinghe & Karunanayake, 2021) based on their main features in terms of predicting a variable response selected, among other applications. Different machine learning techniques were used in this work to find the attributes that most affected admission response behavior in hospitals in the autonomous region of Castilla y León. The analysis was conducted for all data, and each hospital on an individual basis and the algorithms used were:

- Support vector machine (Casalicchio et al., 2018).
- Random forest (Kirasich et al., 2018).
- Logistic regression (Guo et al., 2021).

##### Random Forest as an Attribute Selector Algorithm

This involves a set of decision trees using what is known as the bagging technique to increase generalization capacity and reduce variance in the performance metrics required. They constitute one of the most used algorithms in the industry and are widely applied in determining the importance of attributes. The functioning of this algorithm is based mainly on entropy calculation in Eq. (1) of the data used for each tree and hence used to determine those variables that provide the most information in terms of the classification task.

3 $$\mathrm{Entropy}= -{P}_{i}{log}_{2}({P}_{i})$$

Thus, bagging proposes an algorithmic goal to integrate machine learning algorithms to improve the general performance metrics of the system being used. A block diagram showing the model used is provided below.

**Fig. 5:**
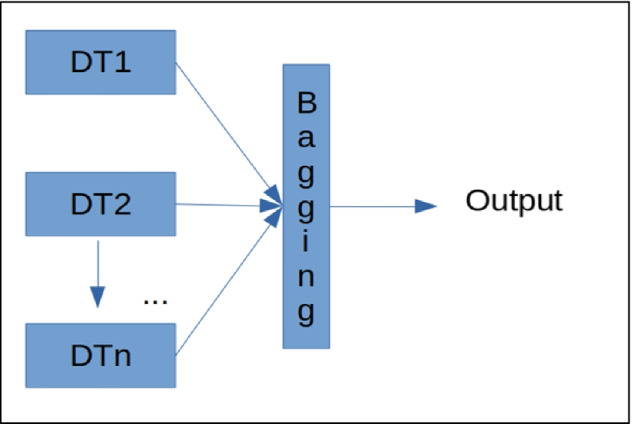
Bagging block or ML

Each ${DT}_{n}$ block corresponds to a decision tree trained using an independent part of the data and is assembled in the last bagging block in inference time to provide a suitably agreed output, as shown in Fig. 5.

##### Multinomial Logistic Regression as an Attribute Selector Algorithm

This is known as a regression technique used to predict a categorical variable. For the purposes of the present work, an attempt was made to predict a patient’s admission to hospital, and thus determine which attributes are the ones that mainly interact to predict them.

Its functioning is based on data analysis according to multinomial distribution, as shown below:

4 $$f\left(x\right)=\frac{n!}{x1!\dots\dots xk!}\;p^{x1}\dots..p^{xk}$$

From this can be obtained a logarithm of the odds ratio or logit, as shown below:

5 $$P^{xi}=exp\;\left(\frac{\frac{Y_i}{N_i}}{X_i}\right)$$

Which represents the attribute incidence of arrangement ${X}_{i}$ in response variable ${Y}_{i}$ making use of the Softmax function shown in Eq. (3), in this polychotomous case. In this specific case, ${Y}_{i}$ corresponds to the dependent or Re_entry and the sets ${X}_{i}$ as described in the “Materials” section.

6 $$\mathrm{Softmax}(x)= \frac{{e}^{xi}}{{\sum }{e}^{xj}}$$

##### Support Vector Machine as an Attribute Selector Algorithm

This constitutes one of the most used algorithms in classification problems and categorical regression. Its simplicity and computational efficiency make it an ideal algorithm for these types of rapid-use and highly reliable applications. Its functioning is based on margin maximization (distance between support vectors of the data being used) to trace a hyperplane that represents the algorithm training stage. In terms of inference, the relative position of the individual vectors is compared to the hyperplane, to thus define the extent to which they belong to the class sets. It may be that model weights will be accessed once the algorithm has been trained.

7 $$H={W}^{T}\times X+B$$

${W}^{T}$ is a vector arrangement whose direction focuses on the solution being sought. Therefore, the importance of the feature can be determined by comparing the size of these coefficients to each other. As such, by observing the SVM coefficients, it is possible to identify the main features used in classification and disregard those deemed unimportant (which are subject to greater variance).

Reducing the number of attributes in machine learning plays a very major role, especially when large datasets are being worked on. Indeed, this can speed up training, prevent overfitting and, ultimately, lead to better classification results thanks to the noise reduction in data.

It is important to highlight the fact that attribute selection is undertaken with the intersection of the variables obtained, i.e., with the common results in the output of the attribute selection algorithms used. It should also be stressed that the implicit order was not taken into account in the case of the algorithm that evidenced the best performance.

##### Attribute Selection with Clinical Meaning by a Clinical Expert

Lastly, the results obtained were shown to the psychiatrist to enable them, through their own knowledge and experience, to determine the validity of the results provided (Bennasar et al., 2015). Thus, the aim was to eliminate any bias caused by the machine learning algorithms and by the data used in the present study.

## Results

In this study, we aimed to analyze those variables that influence admissions in CYL hospitals. The Re_entry variable assumed value 1 when the patient was readmitted to CYL hospitals during the period from 2005 to 2015.

### CHAID Analysis

According to Table 4, in which we showed the classification trees created using CHAID according to hospitals to determine those variables that influence CYL admissions, the reality facing each hospital differs in each region, which is why we shall explain the results found in alphabetical order:

- Avila: The variable explaining admissions (Re_entry) is the *suicidal ideas* variable with chi-square = 22,831; *p* = 0.00 and *df* = 1, accounting for 36% of node 1 with 46.6% of patients being admitted.
- Burgos: Admissions in Burgos according to CHAID are explained by *years*, which are distributed over years before 2008, which is node 1 accounts for 32%; then, admissions in the years 2008 to 2011 are explained by *suicidal ideas* with *p* = 0.03, chi-square = 4,719, and *df* = 1 accounting for 51%, of which 63% of admissions are explained by node 7. Additionally, years after 2011 are accounted for by *personality disorder*, accounting for 38% of admissions according to node 9.
- In the province of Leon, we have data at our disposal from the Leon Welfare Complex (Leon) and El Bierzo Hospital. The CHAID analysis on LeonCA indicate that *depressive syndrome* is the disorder that most influences admissions, with 28%, *p* = 0.001, chi-square = 12,075, and *df* = 1. In the case of Hospital El Bierzo, *gender* proves to be the main variable that has an influence, with *p* = 0.00, chi-square = 13,329, and *df* = 1, after which are *women* in node 2, with *personality disorder* accounting for 44% of admissions with *p* = 0.018, chi-square = 5,594, and *df* = 1 which, according to node 4, accounts for 55% of readmissions.
- In Palencia, a *personality disorder* is a variable that most influences readmissions according to CHAID as shown in Table 4, accounting for 48% with *p* = 0.00, chi-square = 28,161, and *df* = 1 from node 0, and then, 71% of the same cases are accounted for in node 2.
- In Salamanca, the variable with the most influence on readmissions is *gender*, with *p* = 0.006, chi-square = 7,505, and *df* = 1 according to CHAID, accounting for 26% in node 0, and then in node 1 men suffering from *alcohol abuse* would be the next most influential variable with *p* = 0.02, chi-square = 5.378, and *df* = 1.
- In Segovia, the most influential variable accounting for readmissions is *personality disorder*, with *p* = 0.00, chi-square = 34.128, and *df* = 1, accounting for 42% of them according to node 0 from the CHAID analysis.
- In Soria, the most influential variable accounting for readmissions is *adjustment disorder*, with *p* = 0.005, chi-square = 7.958, and *df* = 1, which accounts for 34% according to node 0 from the CHAID analysis.
- In Valladolid, no results were obtained from the CHAID analysis as we only included data from the Valladolid University Hospital, and this failed to produce any result.
- In Zamora, the variable that most influences admissions are *alcohol abuse*, with *p* = 0.00, chi-square = 18.038, and df = 1, accounting for 31% of readmissions.

**Table 4:**
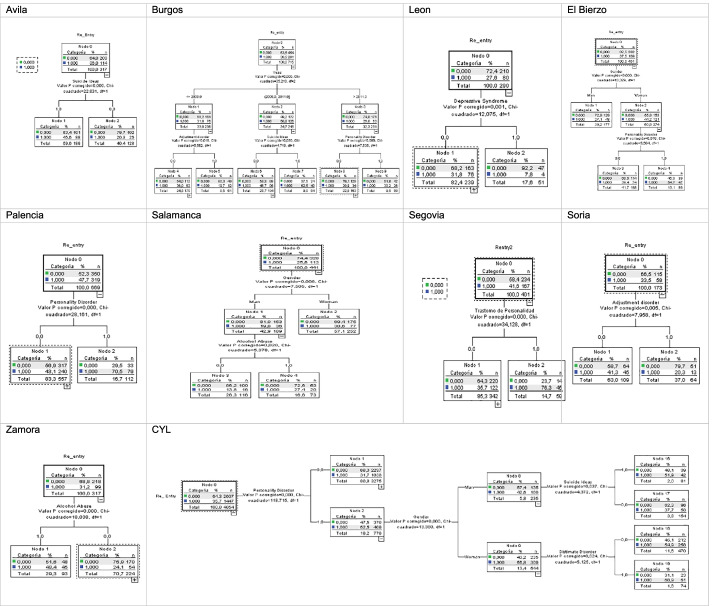
CHAID hospitals

According to Table 4, in CYL, the most influential variable explaining readmissions when combining all hospitals from Figures 1 and 2 was *personality disorder*, with *p* = 0.00, chi-square = 118,715, and *df*=1 accounting for 36% of admissions according to node 0 from the CHAID analysis. Additionally, *gender* in node 2 proves influential in admissions of men in node 6 with *suicidal ideas* accounting for 43%, with *p* = 0.0037, chi-square = 4.372, and *df* = 1, while for women, readmissions are accounted for by node 9, with *dysthymic disorder* accounting for 57% with *p* = 0.024, chi-square = 5.15, and *df* = 1 of readmissions.

In short, the CHAID analysis enables us to rapidly display the most important ratios between variables, thus allowing researchers to recognise and identify profiles—in this case, behavior between 2005 and 2015 at CYL hospitals and how each behaves in terms of readmissions of acute patients with suicide-related mental disorders.

### ML for Feature Selection

#### Correlation Analytics

We shall start this stage of the study by providing the results obtained from the correlation analysis conducted with all the variables using the Pearson and Spearman correlation coefficients in the complementary material of all hospitals included in this study. All the variables explained in Table 2 are compared according to that taken into consideration in the methodology in Figure 3. According to the review by the team of experts, correlation in each subunit of hospital data shows us that the variables do not evidence any correlation with each other, with absolute values above 0.6; i.e., no variable was eliminated from Table 2 owing to correlation.

According to Table 5, we can display those variables that prove most influential in readmissions to CYL hospitals. The analysis was conducted on all data as a whole (CYL), and for each hospital on an individual basis. Different machine learning techniques were applied in this work to find the attributes that most affect behavior regarding response to readmissions associated with mental disorders of acute patients. We can see how each mental disorder variable varies according to region, and how methods or variables that make up suicidal features vary. Generally speaking, the most influential variables for the purpose of this study according to ML in CYL are *adjustment disorder*, *alcohol abuse*, *depressive syndrome*, *personality disorder*, and *dysthymic disorder* in terms of mental disorders; regarding methods, the most influential variables would be *benzodiazepine poisoning*, *suicidal ideas*, *drug poisoning*, *antipsychotic poisoning*, and *suicide and/or self-harm from jumping*. Lastly, we can see how *age* and *gender* influence hospital readmissions.

**Table 5:**
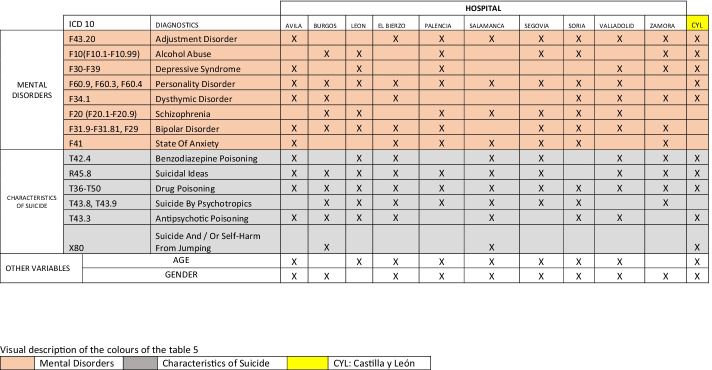
ML hospitals

### Comparison Between CHAID and ML

Table 6 present results of metrics for ML use Splitting 80% Training 20% Testing, organized as follows:

1. A classification model based on 2-layer artificial neuron networks was used, with the following hyperparameters shown in Table 6 that were obtained from grid search for the ML model corresponding to readmissions at all CYL hospitals shown in Tables 5 and 7.
2. A classification model based on kernel methods was used, namely RBF (radial basis functions), with an SVM (support vector machine) activation function as shown in Tables 5 and 7.

**Table 6:** ML metrics for CYL from Tables 5 and 7

Confusion matrix for CYL			Metrics	
	Real		ACC	0.933
Predicted	A	B	Precision	0.927
A	254	20	Recall	0.881
B	34	500	F1 score	0.904
	288	520		
Confusion matrix for CYL			Metrics	
	Real		ACC	0.851
Predicted	A	B	Precision	0.784
A	232	64	Recall	0.805
B	56	456	F1 score	0.794
	288	520		

### Attribute Selection by a Clinical Expert

Assessment or validation of the results shown in Tables 5 and 7 was undertaken by the clinical expert. The expert only pointed out that the variables associated with somatic disorders were not taken into consideration because they are consequences of behavior rather than a cause, whereby they deemed the results associated with mental disorders suitable, taking into account their experience in the field.

**Table 7:**
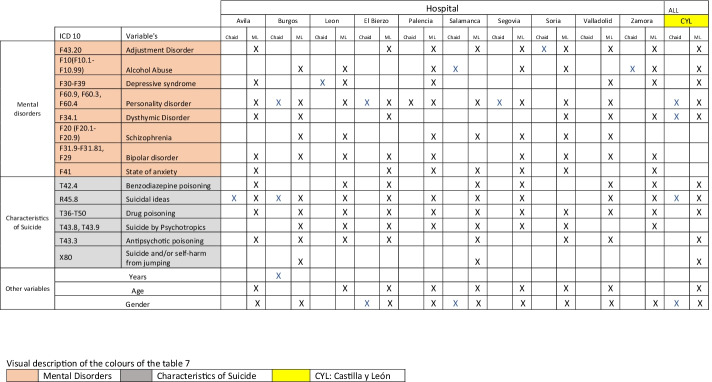
Comparison of results between CHAID and ML according to hospitals

Table 7 shows the results comparing CHAID with ML for each hospital in this region, enabling us to display any coincidences or differences among the variables selected that explain hospital admissions. It is interesting to note that in the case of ML, several variables emerge that are associated with mental disorders and suicidal features. Similarly, the following variables coincide in the case of CHAID and ML: *personality disorder*, *dysthymic disorder*, and *suicidal ideas*, as well as *gender* in the case of the CYL data set as a whole.

It is important to highlight the fact that these algorithms work in different ways, whereby the wide variety of results from each region in addition to the complexity itself that suicide entails, should be understood.

## Discussion

The chi-square analysis is a method that is widely used to identify attributes that have the greatest bearing on accounting for a response variable subject to study. In Pourmand et al. (2021), this method is explained as being used to select attributes based on the statistical distribution of data, although, in many applications, the probability distribution function is unknown and difficult to estimate (Berenfeld & Hoffmann, 2021). That is why a decision was made to use a tree-based iterative method—the CHAID analysis (Jojoa et al., 2021)—complemented by a machine learning-based attributed selection, as the idea was to improve generalisation of the results obtained for these types of clinical issues in which attribute selection is of great importance. Similarly, in (Jojoa-Acosta et al., 2021), the sole use of machine learning methods and assessment by experts is proposed, differing from our work mainly in the recursive selection of attributes and improved with conventional statistical methods, preserving selection by experts in the field to validate outputs of the algorithms used.

Feature selection is an even more challenging task than prediction metrics, which is why there are several methods to carry it out (Gupta et al., 2022). There is a wide field to investigate the use of supervised learning techniques in problems related to stress (Kaur et al., 2022; S. Sharma et al., 2021) and other human behavior disorders using emerging ML techniques (Monga et al., 2022). During COVID-19, ICTs were used more intensively to perform physical and mental monitoring using ML techniques, for example (Pandey et al., 123 C.E.; M. Sharma et al., 2020).

One of the main limitations of the model used is the impossibility of observing the trend in factors in terms of the dependent variable—in this case, hospital admissions. This is the result of the fact that random forest and logistic regression use entropy and probability metrics, respectively, which do not assume any negative values at any time. This in turn limits the conclusions that can be obtained, as those variables that have the greatest bearing on the response variable are identified, albeit not in the contribution direction, i.e., whether it is positive or negative regarding hospital admissions. Furthermore, the importance associated with the order is not taken into account all the time common results are used as the main decision-making rule in the case of the variables selected as being the most important when accounting for hospital admission response.

According to You et al. (2020), policy or strategy-driven interventions should be organized by central governments and regions, considering taking regional features into account to help lower local suicide rates more effectively.

According to Table 7, the variables selected by ML that influence admissions in CYL public hospitals associated with mental disorders are found in the following:

- *Adjustment disorder*, which features in hospitals in Avila, El Bierzo, Palencia, Salamanca, Segovia, Soria, Valladolid, and Zamora. CHAID coincides with the case of the Soria hospital in terms of this disorder. In a study (Fegan & Doherty, 2019), it is suggested that there is a close association between adjustment disorder and suicidal behavior, while in a study (P. Casey et al., 2015), adjustment disorder is associated with suicide as a risk disorder, although the limitations of the study itself did not enable all psychiatric environments to be subject to generalization.
- *Alcohol abuse* is a variable that is selected for hospitals in Burgos, Leon, Palencia, Segovia, Soria, and Zamora as influencing admission of acute patients associated with suicide. CHAID coincides with the case of hospitals in Salamanca and Zamora in terms of this disorder. In a study conducted in Australia, it is maintained that aggressive behavior, comorbidity with other psychiatric disorders and recent interpersonal conflicts such as break-up and family conflicts may lead to suicide in individuals who suffer from alcohol abuse (Kõlves et al., 2017). Research (Conner & Bagge, 2019) indicates that alcohol abuse increases the risk of suicidal behavior, while the use of drugs such as opioids is a close predictor of attempts at suicide (Marengo et al., 2021) in some regions.
- *Depressive syndrome* is an influential variable in the cases of Avila, Leon, Palencia, Valladolid, and Zamora. CHAID coincides with the case of the Leon hospital in terms of this disorder. In this study (Revappala et al., 2021), it turned out that this was the most common disorder among mood disorders involving suicide attempts.
- *Personality disorder* is an influential variable in Avila, Burgos, Leon, El Bierzo, Palencia, Salamanca, Segovia, Soria, and Valladolid. CHAID coincides with the case of the Palencia hospital in terms of this disorder. This study (Doyle et al., 2016) found a 20-fold increase in the risk of suicide among patients with personality disorder in comparison to those without any such recorded psychiatric disorder.
- *Dysthymic disorder* is considered an influential variable in Avila, Burgos, El Bierzo, Soria, Valladolid, and Zamora. The recurring dysthymic disorder would appear to lead to a greater risk of suicide (Witte et al., 2009).
- *Schizophrenia* appears as an influential variable in Burgos, Leon, Palencia, Salamanca, Segovia, Soria, and Valladolid. There is a significant link between schizophrenia and suicide in China (Lyu et al., 2021) and schizophrenic suicides involved a greater intention to commit suicide than those without it (Lyu & Zhang, 2021).
- *Bipolar disorder* is an influential variable in Avila, Burgos, Leon, El Bierzo, Palencia, Salamanca, Segovia, Soria, and Zamora. There have been studies on bipolar disorder and suicide to help understand clinical and demographic factors (Miller & Black, 2020).
- *State of anxiety* is an influential variable in Avila, El Bierzo, Palencia, Segovia, Soria, Valladolid, and Zamora. Mental health diagnoses such as anxiety and/or depression are closely associated with suicide among university students (S. M. Casey et al., 2022).
  According to Table 7, the variables associated with methods or suicidal features selected by ML that influence admissions in CYL public hospitals are found in the following:
- *Benzodiazepine poisoning* is considered an influential variable in Avila, Leon, El Bierzo, Salamanca, Segovia, Valladolid, and Zamora. The high proportion of this type of intentional poisoning, which includes diagnoses of mental health disorders among young women, highlights the importance of assessing mental health and the risk of suicide in emergency services deriving from suitable monitoring, according to this research (Bushnell et al., 2021).
- *Suicidal ideas* are considered an influential variable in Avila, Burgos, Leon, El Bierzo, Palencia, Salamanca, Segovia, Valladolid, and Zamora. CHAID coincides with the case of the hospitals in Avila and Burgos in terms of this variable. In this research (Chapman et al., 2015), it is pointed out that the association of suicidal ideas with subsequent suicide needs to be cautiously interpreted, owing to the great heterogeneous nature of studies and associated disorders.
- *Drug poisoning* is considered an influential variable in Avila, Burgos, Leon, El Bierzo, Palencia, Salamanca, Segovia, Soria, Valladolid, and Zamora. In a cross-sectional study (Shiels et al., 2020), it was found that demographic features and geographic patterns varied according to the cause of death, which suggests that the increase in death results from this cause and alcohol abuse is not merely concentrated within a single group or region.
- *Suicide by psychotropics* is considered an influential variable in Burgos, Leon, El Bierzo, Palencia, Salamanca, Segovia, Soria, and Zamora. There is a study that compares cases of self-intoxication by psychotropics (Pfeifer et al., 2020); the results of which depend on the region subject to study.
- *Antipsychotic poisoning* is considered an influential variable in Avila, Burgos, Leon, El Bierzo, Salamanca, Soria, and Valladolid. In research into this subject (Ferrey et al., 2018), little difference was found in terms of the toxicity of individual mood stabilizers.
- *Suicide and/or self-harm from jumping* is considered an influential variable in Burgos and Salamanca. There is little difference in terms of the features of individuals who jump from different places (Bennewith et al., 2011; Gunnell & Nowers, 1997).
- Of the other variables considered influential according to Table 7, only Burgos showed via the CHAID analysis that the *years* variable accounted for its admission behavior; in the case of the other hospitals, this variable was not selected according to the methodology proposed in the study. Specifically, the economic crisis (Mattei et al., 2019) and the increase in unemployment (Chang et al., 2013; López-Contreras et al., 2019) are considered important risk factors regarding suicide (Demirci et al., 2020). In general, it is estimated that this is accentuated in situations of economic uncertainty (Vandoros et al., 2019), or when the situation regarding family poverty worsens, especially if associated with previous mental health problems (Pan et al., 2013). Consequently, and considering that a situation of world trade collapse is being announced leading to a major economic crisis as a result of the pandemic (Slater, 2020), this will foreseeably influence suicide rates, as occurs in the case of all disasters (Mannix et al., 2020).
- *Age* is another variable considered influential in the case of hospitals in Avila, Leon, El Bierzo, Palencia, Salamanca, Segovia, Soria, and Valladolid, according to the results obtained in Table 5. In this study (Da Veiga & Saraiva, 2003), the practical implications within the context of previous theories that relate suicide age patterns to sociological and economic dimensions are discussed.
- *Gender* is a further variable considered influential in the case of hospitals in Avila, Burgos, El Bierzo, Palencia, Salamanca, Segovia, Soria, Valladolid, and Zamora, according to the results obtained by ML in Table 7. The CHAID analysis also coincides with the El Bierzo and Salamanca hospitals in terms of this variable. Considering the differences in intention to commit suicide between men and women highlighted in this study (Freeman et al., 2017), gender-oriented prevention and intervention strategies would be recommended, whereby this variable proves to be influential in accounting for admissions of acute patients associated with suicide.

### Limitation

DBSUICIDECYL contains the records of acute patients with suicide-related mental disorders and represents one of the most diverse cohorts in the country. The nature of this study also limits the records of patients meeting the inclusion criteria shown in Table 2.

The data analyzed here has been anonymised (BDSUICIDECYL), and as such, there is no knowledge of the patient’s socio-economic data. However, the period from which the data was taken was from 2005 to 2015, including the 2008 period of the financial crisis. According to the studies by Roca et al. (2013), to assess the relationship between suicide and the economic crisis, we must avoid focusing on immediate suicide rates, but rather, first look at the underlying diseases and only later at the consequences of those diseases, i.e. suicide, and the use of health services. That is why we focused on mental disorders and suicidal features when carrying out this study.

## Conclusion

The relevance of this study is to show the variables for each hospital and for the entire region with their metrics in order to show the feature selection that allow us to understand patients who are readmitted with suicidal behavior.

According to an ML analysis on CYL hospital readmissions of acute patients with suicide-related mental disorders between 2005 and 2015, we found variables that influence *adjustment disorder*, *alcohol abuse*, *depressive syndrome*, *personality disorder*, and *dysthymic disorder*.

Of the methods or features associated with suicide over the same period, in the ML analysis carried out, we found influential variables such as *benzodiazepine poisoning*, *suicidal ideas*, *drug poisoning*, *antipsychotic poisoning*, and *suicide*, and/or *self-harm from jumping*. Other influential variables that we found in this study were *age* and *gender*.

For its part, the CHAID analysis coincided with ML in variables influencing *personality disorder*, *gender*, *suicidal ideas*, and *dysthymic disorder.*

According to the results obtained, it is necessary to continue investigating the various factors that affect suicide; in this case, we address the hospital management of readmissions. However, there are other areas of research that can contribute to suicide prevention. All of them contribute to the knowledge of this problem, for example, we can mention initiatives that help prevent it, such as training activities for its professionals (Castillo-Sanchez et al., 2019) and mindfulness therapies in times of COVID (Castillo-Sánchez et al., 2022).

Expected future work will involve verifying suicide-related mental disorders over the years 2016 to 2020 in the same region.

## References

[CR1] Bennasar M, Hicks Y, Setchi R (2015). Feature selection using Joint Mutual Information Maximisation. Expert Systems with Applications.

[CR2] Bennewith O, Nowers M, Gunnell D (2011). Suicidal behaviour and suicide from the Clifton Suspension Bridge, Bristol and surrounding area in the UK: 1994–2003. European Journal of Public Health.

[CR3] Berenfeld, C., & Hoffmann, M. (2021). Density estimation on an unknown submanifold. 10.1214/21-EJS1826, *15*(1), 2179–2223. 10.1214/21-EJS1826,15(1),2179-2223.10.1214/21-EJS1826

[CR4] Bushnell GA, Olfson M, Martins SS (2021). Sex differences in US emergency department non-fatal visits for benzodiazepine poisonings in adolescents and young adults. Drug and Alcohol Dependence.

[CR5] Casalicchio G, Molnar C, Bischl B (2018). Visualizing the feature im portance for black box models. Lecture Notes in Computer Science (Including Subseries Lecture Notes in Artificial Intelligence and Lecture Notes in Bioinformatics).

[CR6] Casey P, Jabbar F, O’Leary E, Doherty AM (2015). Suicidal behaviours in adjustment disorder and depressive episode. Journal of Affective Disorders.

[CR7] Casey SM, Varela A, Marriott JP, Coleman CM, Harlow BL (2022). The influence of diagnosed mental health conditions and symptoms of depression and/or anxiety on suicide ideation, plan, and attempt among college students: Findings from the Healthy Minds Study, 2018–2019. Journal of Affective Disorders.

[CR8] Castillo-Sánchez G, Sacristán-Martín O, Hernández MA, Muñoz I, De La Torre I, Franco-Martín M (2022). Online mindfulness experience for emotional support to healthcare staff in times of Covid-19. Journal of Medical Systems.

[CR9] Castillo-Sanchez, G. A., De La Torre Diez, I., Rodrigues, J. J. P. C., Munoz-Sanchez, J. L., Hernandez-Ramos, A., & Franco, M. A. (2019). Development of an E-learning model for training health staff in suicide prevention. In IEEE (Ed.), *2019 IEEE International Conference on E-Health Networking, Application and Services, HealthCom 2019* (pp. 1–16). Institute of Electrical and Electronics Engineers Inc. 10.1109/HealthCom46333.2019.9009599

[CR10] Chang SS, Stuckler D, Yip P, Gunnell D (2013). Impact of 2008 global economic crisis on suicide: Time trend study in 54 countries. BMJ (online).

[CR11] Chapman CL, Mullin K, Ryan CJ, Kuffel A, Nielssen O, Large MM (2015). Meta-analysis of the association between suicidal ideation and later suicide among patients with either a schizophrenia spectrum psychosis or a mood disorder. Acta Psychiatrica Scandinavica.

[CR12] Conner KR, Bagge CL (2019). Suicidal behavior: Links between alcohol use disorder and acute use of alcohol. Alcohol Research: Current Reviews.

[CR13] Da Veiga FA, Saraiva CB (2003). Age patterns of suicide: Identification and characterization of European clusters and trends. Crisis.

[CR14] Demirci Ş, Konca M, Yetim B, İlgün G (2020). Effect of economic crisis on suicide cases: An ARDL bounds testing approach. International Journal of Social Psychiatry.

[CR15] Doyle M, While D, Mok PLH, Windfuhr K, Ashcroft DM, Kontopantelis E, Chew-Graham CA, Appleby L, Shaw J, Webb RT (2016). Suicide risk in primary care patients diagnosed with a personality disorder: A nested case control study. BMC Family Practice.

[CR16] Fegan, J., & Doherty, A. M. (2019). Adjustment disorder and suicidal behaviours presenting in the general medical setting: A systematic review. In *International Journal of Environmental Research and Public Health,* *16*(16),MDPI AG. 10.3390/ijerph16162967 10.3390/ijerph16162967 PMC6719096 31426568

[CR17] Ferrey AE, Geulayov G, Casey D, Wells C, Fuller A, Bankhead C, Ness J, Clements C, Gunnell D, Kapur N, Hawton K (2018). Relative toxicity of mood stabilisers and antipsychotics: Case fatality and fatal toxicity associated with self-poisoning. BMC Psychiatry.

[CR18] Freeman A, Mergl R, Kohls E, Székely A, Gusmao R, Arensman E, Koburger N, Hegerl U, Rummel-Kluge C (2017). A cross-national study on gender differences in suicide intent. BMC Psychiatry.

[CR19] Ghosh, A., Nashaat, M., Miller, J., & Quader, S. (2021). Context-based evaluation of dimensionality reduction algorithms—Experiments and statistical significance analysis. *ACM Transactions on Knowledge Discovery from Data (TKDD)*, *15*(2). 10.1145/3428077

[CR20] Gunnell D, Nowers M (1997). Suicide by jumping. Acta Psychiatrica Scandinavica.

[CR21] Guo Y, Zhang Z, Tang F (2021). Feature selection with kernelized multi-class support vector machine. Pattern Recognition.

[CR22] Gupta, R., Shrivas, A., & Shukla, R. (2022). A two-stage multifeature selection method to predict healthcare data using neural network. *EAI/Springer Innovations in Communication and Computing*, 77–87. 10.1007/978-3-030-78284-9_4/COVER/

[CR23] Haw C, Hawton K (2015). Suicide is a complex behaviour in which mental disorder usually plays a central role. Australian and New Zealand Journal of Psychiatry.

[CR24] INE. (2021). *Deaths by death’s cause in Spain - 2020*.

[CR25] Invest in Spain. (n.d.). *Industrias destacadas*. Retrieved October 8, 2021, from https://www.investinspain.org/es/regiones/castilla-y-leon/industrias-destacadas

[CR26] Jojoa M, Lazaro E, Garcia-Zapirain B, Gonzalez MJ, Urizar E (2021). The impact of COVID 19 on university staff and students from Iberoamerica: Online learning and teaching experience. International Journal of Environmental Research and Public Health.

[CR27] Jojoa-Acosta MF, Signo-Miguel S, Garcia-Zapirain MB, Gimeno-Santos M, Méndez-Zorrilla A, Vaidya CJ, Molins-Sauri M, Guerra-Balic M, Bruna-Rabassa O (2021). Executive functioning in adults with down syndrome: Machine-learning-based prediction of inhibitory capacity. International Journal of Environmental Research and Public Health 2021.

[CR28] Kaur P, Gautam R, Sharma M (2022). Feature selection for bi-objective stress classification using emerging swarm intelligence metaheuristic techniques. Lecture Notes on Data Engineering and Communications Technologies.

[CR29] Kirasich, K., Smith, T., & Sadler, B. (2018). Random forest vs logistic regression: Binary classification for heterogeneous datasets. *SMU Data Science Review*, *1*(3). https://scholar.smu.edu/datasciencereview/vol1/iss3/9

[CR30] Kõlves K, Draper BM, Snowdon J, De Leo D (2017). Alcohol-use disorders and suicide: Results from a psychological autopsy study in Australia. Alcohol (Fayetteville NY).

[CR31] Llanes-Álvarez C, Alberola-López C, Andrés-de-Llano JM, Álvarez-Navares AI, Pastor-Hidalgo MT, Roncero C, Garmendia-Leiza JR, Franco-Martín MA (2021). Hospitalization trends and chronobiology for mental disorders in Spain from 2005 to 2015. Chronobiology International.

[CR32] Llanes-Álvarez, C., Andrés-de Llano, J. M., Álvarez-Navares, A. I., Pastor-Hidalgo, M. T., Roncero, C., & Franco-Martín, M. A. (2020). Trends in psychiatric hospitalization for alcohol and drugs in Castilla y León between 2005 and 2015. *Adicciones*, *0*(0). 10.20882/ADICCIONES.1405 10.20882/adicciones.1405 33338242

[CR33] López-Contreras N, Rodríguez-Sanz M, Novoa A, Borrell C, Medallo Muñiz J, Gotsens M (2019). Socioeconomic inequalities in suicide mortality in Barcelona during the economic crisis (2006–2016): A time trend study. British Medical Journal Open.

[CR34] Lyu J, Zhang J (2021). Suicide means, timing, intent and behavior characteristics of the suicides with schizophrenia. Psychiatry Research.

[CR35] Lyu J, Zhang J, Hennessy DA (2021). Characteristics and risk factors for suicide in people with schizophrenia in comparison to those without schizophrenia. Psychiatry Research.

[CR36] Mannix R, Lee LK, Fleegler EW (2020). Coronavirus disease 2019 (COVID-19) and firearms in the United States: Will an epidemic of suicide follow?. Annals of Internal Medicine.

[CR37] Marengo L, Douaihy A, Zhong Y, Krancevich K, Brummit B, Sakolsky D, Deal M, Zelazny J, Goodfriend E, Saul M, Murata S, Thoma B, Mansour H, Tew J, Ahmed N, Marsland A, Brent D, Melhem NM (2021). Opioid use as a proximal risk factor for suicidal behavior in young adults. Suicide and Life-Threatening Behavior.

[CR38] Mattei G, Pistoresi B, De Vogli R (2019). Impact of the economic crises on suicide in Italy: The moderating role of active labor market programs. Social Psychiatry and Psychiatric Epidemiology.

[CR39] Melendez frigola, C., Arroyo Borrell, E., & Saez, M. (2016). Data analysis of sub-acute patients with information registered in the minimum basic set of social health data (cmbd). *Rev Esp Salud Pública*, *90*(3), e1–e7. https://www.mscbs.gob.es/biblioPublic/publicaciones/recursos_propios/resp/revista_cdrom/VOL90/ORIGINALES/RS90C_CMF.pdf27708254

[CR40] Miller JN, Black DW (2020). Bipolar disorder and suicide: A review. Current Psychiatry Reports 2020.

[CR41] Moitra M, Santomauro D, Degenhardt L, Collins PY, Whiteford H, Vos T, Ferrari A (2021). Estimating the risk of suicide associated with mental disorders: A systematic review and meta-regression analysis. Journal of Psychiatric Research.

[CR42] Monga, P., Sharma, M., & Sharma, S. K. (2022). *Performance analysis of machine learning and soft computing techniques in diagnosis of behavioral disorders*. 85–99. 10.1007/978-981-16-9488-2_8

[CR43] Munasinghe K, Karunanayake P (2021). Recursive feature elimination for machine learning-based landslide prediction models. 3rd International Conference on Artificial Intelligence in Information and Communication.

[CR44] Pan YJ, Stewart R, Chang CK (2013). Socioeconomic disadvantage, mental disorders and risk of 12-month suicide ideation and attempt in the National Comorbidity Survey Replication (NCS-R) in US. Social Psychiatry and Psychiatric Epidemiology.

[CR45] Pandey, R., Gautam, V., Pal, R., Bandhey, H., Singh Dhingra, L., Misra, V., Sharma, H., Jain, C., Bhagat, K., Patel, L., Agarwal, M., Agrawal, S., Jalan, R., Wadhwa, A., Garg, A., Agrawal, Y., Rana, B., Kumaraguru, P., & Sethi, T. (123 C.E.). *A machine learning application for raising WASH awareness in the times of COVID-19 pandemic*. 10.1038/s41598-021-03869-6 10.1038/s41598-021-03869-6 PMC8764038 35039533

[CR46] Pfeifer P, Greusing S, Kupferschmidt H, Bartsch C, Reisch T (2020). A comprehensive analysis of attempted and fatal suicide cases involving frequently used psychotropic medications. General Hospital Psychiatry.

[CR47] Pourmand S, Shabbak A, Ganjali M (2021). Feature selection based on divergence functions: A comparative classiffication study. Statistics Optimization and Information Computing.

[CR48] Qasim OS, Algamal ZY (2018). Feature selection using particle swarm optimization-based logistic regression model. Chemometrics and Intelligent Laboratory Systems.

[CR49] Revappala, B. C., Mallanaik, S., Vijayakumar, V. K., Kudumallige, S. K., & Eshwarappa, S. N. (2021). Prevalence of psychiatric comorbidity among suicide attempters. *Journal of Evolution of Medical and Dental Sciences*, *10*(38), 3370–3374. https://go.gale.com/ps/i.do?p=AONE&sw=w&issn=22784748&v=2.1&it=r&id=GALE%7CA677900954&sid=googleScholar&linkaccess=fulltext

[CR50] Roca, M., Gili, M., Garcia-Campayo, J., & García-Toro, M. (2013). Economic crisis and mental health in Spain. In The Lancet 382(9909):pp. 1977-1978. Elsevier B.V. 10.1016/S0140-6736(13)62650-1 10.1016/S0140-6736(13)62650-1 24332073

[CR51] Sacyl. (2021). *CYL health*. SACYL. https://www.saludcastillayleon.es/en

[CR52] Sharma S, Singh G, Sharma M (2021). A comprehensive review and analysis of supervised-learning and soft computing techniques for stress diagnosis in humans. Computers in Biology and Medicine.

[CR53] Sharma, M., Sharma, S., & Singh, G. (2020). Remote monitoring of physical and mental state of 2019-nCoV victims using social internet of things, fog and soft computing techniques. *Computer Methods and Programs in Biomedicine*, *196*. 10.1016/J.CMPB.2020.105609 10.1016/j.cmpb.2020.105609 32593062

[CR54] Shiels, M. S., Tatalovich, Z., Chen, Y., Haozous, E. A., Hartge, P., Nápoles, A. M., Pérez-Stable, E. J., Rodriquez, E. J., Spillane, S., Thomas, D. A., Withrow, D. R., Berrington De González, A., & Freedman, N. D. (2020). Trends in mortality from drug poisonings, suicide, and alcohol-induced deaths in the United States from 2000 to 2017. *JAMA Network Open*, *3*(9). 10.1001/JAMANETWORKOPEN.2020.16217 10.1001/jamanetworkopen.2020.16217 PMC7489841 32915234

[CR55] Slater, A. (2020). *Coronavirus is crushing world trade*. https://resources.oxfordeconomics.com/hubfs/OE-Downloads/0000027.pdf?utm_campaign=Promotional Campaigns-UK&utm_medium=email&_hsmi=83701646&_hsenc=p2ANqtz--73WTERBoGeFt0o4lIdVu3TETsSqeZgpSA6qJvk1IU_kxcBSQlUhGqvGg7f3_TBbSOyFQy&utm_content=83701646&utm_source=hs_automation

[CR56] Spain, government of. (2021). *eCIE-Maps - CIE-10-ES Diagnósticos*. Diagnosticos. https://eciemaps.mscbs.gob.es/ecieMaps/browser/index_10_mc.html

[CR57] Too LS, Spittal MJ, Bugeja L, Reifels L, Butterworth P, Pirkis J (2019). The association between mental disorders and suicide: A systematic review and meta-analysis of record linkage studies. Journal of Affective Disorders.

[CR58] Vandoros S, Avendano M, Kawachi I (2019). The association between economic uncertainty and suicide in the short-run. Social Science and Medicine.

[CR59] Wan Y, Li T, Wang P, Duan S, Zhang C, Li N (2021). Robust and efficient classification for underground metal target using dimensionality reduction and machine learning. IEEE Access.

[CR60] Wang, Z., Li, H., Nie, B., Du, J., Du, Y., & Chen, Y. (2021). Feature selection using different evaluate strategy and random forests. *2021 International Conference on Computer Engineering and Artificial Intelligence (ICCEAI)*, 310–313. 10.1109/ICCEAI52939.2021.00062

[CR61] Williams SC, Schmaltz SP, Castro GM, Baker DW (2018). Incidence and method of suicide in hospitals in the United States. The Joint Commission Journal on Quality and Patient Safety.

[CR62] Witte TK, Timmons KA, Fink E, Smith AR, Joiner TE (2009). Do major depressive disorder and dysthymic disorder confer differential risk for suicide?. Journal of Affective Disorders.

[CR63] You BS, Jeong KH, Cho HJ (2020). Regional suicide rate change patterns in Korea. International Journal of Environmental Research and Public Health.

